# Retrospective Clinical Evaluation of Subgingival Composite Resin Restorations with Deep-Margin Elevation

**DOI:** 10.3290/j.jad.b3240665

**Published:** 2022-08-19

**Authors:** Clara Muscholl, Nadja Zamorska, Kyrill Schoilew, Caroline Sekundo, Christian Meller, Christopher Büsch, Diana Wolff, Cornelia Frese

**Affiliations:** a Dentist, Department of Conservative Dentistry, Clinic for Oral, Dental, and Maxillofacial Diseases, Heidelberg University Hospital, Heidelberg, Germany. Examined patients, data collection, wrote the manuscript.; b Student, Department of Conservative Dentistry, Periodontology and Endodontology, University Center of Dentistry, Oral Medicine and Maxillofacial Surgery, Tübingen University Hospital, Tübingen, Germany. Examined patients.; c Lecturer and Dentist, Department of Conservative Dentistry, Clinic for Oral, Dental, and Maxillofacial Diseases, Heidelberg University Hospital, Heidelberg, Germany. Study conception and design.; d Lecturer and Dentist, Department of Conservative Dentistry, Clinic for Oral, Dental, and Maxillofacial Diseases, Heidelberg University Hospital, Heidelberg, Germany. Interpretation of results.; e Lecturer and Dentist, Department of Conservative Dentistry, Periodontology and Endodontology, University Center of Dentistry, Oral Medicine and Maxillofacial Surgery, Tübingen University Hospital, Tübingen, Germany. Examined patients.; f Statistical Scientist, Institute of Medical Biometry, University of Heidelberg, Heidelberg, Germany. Performed statistical analysis.; g Professor, Department of Conservative Dentistry, Clinic for Oral, Dental, and Maxillofacial Diseases, Heidelberg University Hospital, Heidelberg, Germany. Study idea, study conception and design.; † These authors contributed equally to this work.

**Keywords:** proximal box elevation, subgingival defects, gingival and periodontal inflammation, resin composite restoration.

## Abstract

**Purpose:**

To evaluate the long-term clinical quality of subgingivally placed composite resin restorations and the inflammatory status of surrounding supracrestal gingival and periodontal tissues.

**Materials and Methods:**

Patients with at least one subgingival restoration with deep-margin elevation placed between 2010 and 2020 at Heidelberg University Hospital and Tübingen University Hospital were identified. A sound tooth was used as control. Intraoral examination including probing depth (PD), clinical attachment level (CAL), bleeding on probing (BOP), gingival bleeding index (GBI), and plaque control record (PCR) was conducted. The clinical quality of the restorations was evaluated using the modified FDI criteria. For comparison between control and test teeth, a logistic mixed-effects model was used for GBI, PCR, and BOP, while a linear mixed-effects model was used for CAL. Multivariable linear and logistic regressions were used to examine the influence of smoking, age of restoration, number of decayed, missing and filled teeth, use of interdental brushes, and CAL.

**Results:**

Sixty-three patients were included in the study. The mean age of the restorations was 2.70 ± 1.90 years. There were no significant differences between test and control teeth with respect to inflammatory parameters BOP, GBI, and PCR. CAL was significantly higher in test teeth than in controls (p = 0.027). The regression models revealed that CAL has a significant influence on GBI (p = 0.008) and BOP (p < 0.001). A significantly increased GBI occurred especially on test teeth in patients who did not use interdental brushes daily (p = 0.010). The clinical quality of restorations was rated excellent or good in 70%, an no restoration was rated unacceptable.

**Conclusion:**

In this study, no increased inflammation was observed on sites with subgingivally placed composite restorations over an observation period of approximately 3 years. Regular interdental brush use was associated with less gingival inflammation.

In everyday clinical work, the practitioner is frequently confronted with extensive deep subgingival defects adjacent to the alveolar bone crest. Not only does the restoration of these defects present a technical challenge, but the supracrestal tissue attachment,^24^ previously known as biological width,^45^ must also be taken into consideration. The more recent term more accurately describes this area histologically, as it consists of the junctional epithelium and supracrestal connective tissue attachment.^24^ When restoring subgingival defects, the supracrestal tissue attachment can be violated, possibly leading to indication for surgical crown lengthening or orthodontic extrusion of the tooth^9,21,40,42^ in order to avoid chronic gingivitis, periodontal attachment loss, or alveolar bone resorption.^31,34,35^ However, the dimensions of the supracrestal tissue attachment are not constant^45^ and can vary, depending on the position of the tooth, the tooth surface,^27^ the biotype of the gingiva,^7^ and the type of alveolar bone.^2,14,39,46^ Provided that the dentist is able to place the restoration margins in the subgingival area smoothly and without marginal irritation, chronic inflammation and loss of the alveolar bone can most likely be avoided.^19^

Various approaches using different material groups (glass-ionomer cements, composites) for the treatment of deep subgingival defects which extend below the cemento-enamel junction are described in the literature.^12,25,32^ All concepts are based on a two-step approach which includes an elevation of the deep margin in the first step. This deep-margin elevation (DME) allows the proximal margin to be raised to the supragingival level. In the second step, either a direct restoration^19^ or an indirect restoration – a so-called hybrid restoration – can be made.^17^

In general, composite restorations below the cemento-enamel junction show higher failure rates than composite restorations with supragingival margins,^28^ which is probably due to technical difficulties such as isolation, moisture control, and insufficient light polymerization in the subgingival area. Several in-vitro studies on DME are available, focusing on the marginal quality and microleakage.^25,26^ In addition, in-vitro studies have been done on the fracture strength of CAD/CAM ceramic inlays in hybrid restorations after DME.^5,48^ With respect to marginal quality and microleakage, most of the previously published papers on deep restorations were in-vitro studies focusing on the comparison of marginal adaptation between indirect restorations with and without DME.^18,33,41,45,51^ As a result, they found that there were no significant differences in marginal quality between subgingivally cemented indirect restorations and restorations with previous DME.^25,26^

For many years, the clinical quality of two-step restorations by means of DME was only described in clinical case reports.^19^ In 2018, a systematic review^25^ identified seven in-vitro studies and five clinical reports on the indirect restoration of deep subgingival defects with previous DME. The analyzed laboratory studies mainly focused on different composite materials and bonding agents used for DME prior to the final indirect restoration and their influence on marginal adaptation, fracture behavior, and bond strength.^8,18,23,33,41,45,51^ The authors^25^ suggested that those characteristics might be compromised in a clinical setting, since inadequate sealing in deep cavities may lead to diminished margin quality. The case reports found provided clinical documentation and description of treatment protocols,^11,26,30,47^ but no further clinical studies could be identified. The evidence was therefore not considered sufficient for or against a clinical recommendation for DME in subgingival cavities.^25^

More recently, three clinical studies investigating the effects of deep subgingival restorations on periodontal health^3,17^ and their 10-year survival rate^4^ were published. Although those studies provided promising results, further clinical investigations with larger study populations and long-term recalls are needed. In addition, although most studies assessed hybrid restorations (DME with composite resin and subsequent indirect restoration), studies on two-phase composite resin restorations with DME are lacking.

The aim of this retrospective study was therefore to evaluate the long-term clinical quality and gingival and periodontal response of direct composite restorations subgingivally placed by means of DME.

## MATERIALS AND METHODS

### Study Population

This study was conducted at the Department of Conservative Dentistry at Heidelberg University Hospital and the Department of Conservative Dentistry at Tübingen University Hospital. The study protocol was approved by the local ethics committees of the Heidelberg Medical Faculty (protocol no. S-053/2018) and the Tübingen Medical Faculty (protocol no. 522/2019BO2). This study followed the Strengthening the Reporting of Observational Studies in Epidemiology (STROBE) guidelines.^50^

From 2019 to 2020, patients were identified who had received a composite resin restoration in conjunction with a DME between 2010 and 2020, and had at least one caries-free tooth with no restoration in contact with the gingival margin. All restorations had been performed by well-trained practitioners at Heidelberg University Hospital or Tübingen University Hospital following the treatment protocol described below. Inclusion criteria were as follows: patients 18 years or older; patients in good general health; patients with the ability to perform a proper daily oral hygiene regime by themselves. Excluded from the study were: pregnant or lactating women; patients with periodontitis requiring treatment; patients who had been treated with antibiotics within 3 months prior to the study examination; patients who currently required antibiotic prophylaxis. Patients were screened for periodontitis and excluded if any untreated periodontal condition was present. However, those who had received periodontal treatment and attended supportive periodontal therapy were included in the study. Examinations took place at both centers in Heidelberg and Tübingen between 2019 and 2020 and were each performed by one examiner. In view of sample sizes reached by previous studies and for the sake of feasibility, a sample size of 60 participants was set. Assuming a significance level of α = 0.05 and a power of 0.8, this sample size results in an estimated effect of d = 0.32, demonstrating the study’s ability to observe a medium effect size^6^ between the two groups.

### Treatment Protocol

**Fig 1:**
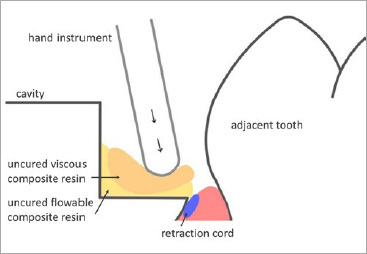
Schematic drawing of the snowplow technique in the first phase of DME.

The examined direct resin composite restorations of all extensive deep-margin cavities were placed using a two-step restoration technique that has been described in literature previously.^19^ After caries removal, the cavity is first “idealized” by elevating the proximal cavity floor and, if necessary, part of the buccal and oral defect walls is built up without a matrix. Due to the deep subgingival cavity margin, the application of rubber-dam is not possible, so moisture control is performed using cotton rolls and suction. In addition, strict bleeding control via retraction cords and astringent agents (Astringedent X, Ultradent; South Jordan, UT, USA) is indicated. After applying a 3-step etch-and-rinse adhesive (Optibond FL, Kerr; Orange, CA, USA), composite resin (Tetric Evo Ceram and Tetric Evo Flow; Ivoclar Vivadent, Schaan, Liechtenstein) is applied using the “snowplow technique”:^38^ a small amount of flowable composite is applied into the cavity and carefully adapted to the cavity floor without light curing. Viscous restorative composite is then placed on the uncured flowable material and modeled in. The flowable composite is displaced by the more viscous material into all areas of the proximal box (Fig 1). This special technique allows achieving excellent homogeneous distribution of the composite resin materials and marginal adaptation. However, it is important to mention that a high level of difficulty is inherent to this technique, and especially marginal excesses should be removed thoroughly before light curing. After light curing, DME should be checked for composite overhangs along the margins, which must be removed carefully to avoid chronic inflammation and periodontal irritation. After completion, marginal quality is ascertained using a dental probe.

In the second step, rubber-dam isolation is now possible due to the idealization of the cavity in step one, culminating in a dry working area. In addition, an anatomically pre-contoured partial matrix (Palodent, Dentsply Sirona; Konstanz, Germany) with wedge and separation ring is used to shape an anatomical crown morphology and a tight proximal contact. The previously placed DME is cleaned carefully and roughened with 50-µm Al_2_O_3_ powder (Kaltenbach & Voigt; Biberach, Germany). The second restoration is placed using the same materials as previously described. For finishing the restoration, the Astropol finishing Kit (Astropol HP, Ivoclar Vivadent) is used. At the end of the treatment session, the selection of accurately fitting interdental brushes and oral hygiene instructions are essential. The accuracy of fit of the interdental brushes should be re-evaluated during the subsequent prophylaxis sessions.

### Clinical Examination

The clinical examination of this study comprised a detailed anamnesis and a questionnaire, an evaluation of the clinical quality of the subgingival restorations according to the modified FDI criteria,^22^ the periodontal condition of the treated and the control teeth (probing depth [PD] and clinical attachment level [CAL]), as well as gingival and periodontal indices.^1,37^

In the questionnaire, each patient was asked about their health status, smoking habits, and their daily oral hygiene, including the usage of interdental brushes. The intraoral examination was performed using mirror and probe, and magnifying loupes (2.5X) with an additional light source. To quantify the individual’s caries experience, the Decayed, Missing and Filled Teeth index (DMF-T) was taken.^50^ Prior to performing the examinations, all clinical investigators participated in a web-based training and calibration (www.e-calib.info) session regarding the FDI criteria for clinical quality assessment, as suggested by Hickel et al.^22^ No calibration was performed regarding other clinical parameters.

### Gingival and Periodontal Condition

To assess periodontal health, PD and CAL were measured at the proximal region of the restorations using a periodontal probe (PCPUNC 15, Hu-Friedy; Chicago, IL, USA). Bleeding on probing (BOP) at those locations was documented dichotomously (“BOP: yes”; “BOP: no”). To asses gingival inflammation, the gingival bleeding index was recorded.^1^ Any bleeding was documented binarily (“gingival bleeding: yes”; ”gingival bleeding: no”) at the target sites of the test and control teeth.

The plaque control record (PCR) was recorded using a plaque indicator liquid (Mira-2-Ton Liquid, Hager & Werken; Duisburg, Germany) in a binary manner (“dental plaque: yes”; “dental plaque: no”) at the relevant target sites of test and control teeth.^37^

### Clinical Quality Criteria

The clinical quality of the resin composite restorations was evaluated using the modified FDI criteria.^22^ These comprise the following categories: esthetic properties (surface luster, surface staining, color stability/translucency, and anatomic form), functional properties (fractures/retention, marginal adaptation, wear, and patients’ view), and biological properties (postoperative [hyper-]sensitivity/tooth vitality, recurrence of caries/erosion/abfraction, and tooth and periodontal response). The category “cleanability” was only assessed for subjects who were examined at Heidelberg University Hospital. This evaluation provides ordinally structured data for the outcome variables (1 = excellent result; 2 = good result; 3 = acceptable result; 4 = reparation of the restoration necessary for prevention; 5 = unacceptable, replacement required).

### Statistical Analysis

Continuous variables were expressed as mean ± SD and categorical variables as absolute and relative frequencies. Appropriate testing of distribution of continuous variables and the corresponding tests (Student’s t-test or Mann-Whitney U-test) were performed. For categorical variables, Pearson’s chi-squared test was used.

To compare the parameters GBI, BOP, PCR, and CAL between the test and control teeth, logistic or linear mixed models were performed if the outcome was nominal or continuous, respectively. Since for each patient one control and one test tooth were analyzed, multiple measurements per patient (paired data) were available. Hence, a random patient-effect term was added to the model to account for patient heterogeneity. Furthermore, periodontitis (yes/no), group (test/control), and an interaction effect between group and periodontitis status were added to the models as independent variables. Furthermore, to adjust for possible center heterogeneity, the center (Heidelberg/Tübingen) was added to the models as an adjustment covariate. However, the mixed-effect model that was implemented for the outcome GBI did not converge, due to a small sample size in combinations of categorical variables. Consequently, in this case, the McNemar test was used for comparison of the groups (test/control teeth).

To investigate influencing factors of possible infections in restorations as measured by GBI, BOP, and PCR, linear mixed-effect models were constructed using only the test teeth (restorations). For these three models, the following independent variables were considered: smoking status (yes/no), age of restoration (in years), DMFT, CAL (mean of all measurements per patient), usage of interdental brushes (yes/no). Furthermore, to adjust for possible center heterogeneity, the center (Heidelberg/Tübingen) was added to the models as an adjustment covariate.

Statistical analyses were conducted using the statistics software R (version 4.0.2, R Core Team; Auckland, New Zealand) using the packages “lme4” and “lmerTest” for linear and logistic mixed effect models and “ggplot” for data illustrations and G*Power, Version 3.1.^15^

## RESULTS

### General Data

The results are presented in Table 1. A total of 63 patients with at least one subgingival restoration with DME placed between 2010 and 2018 (test tooth) were included in this study. Thirty-nine (61.9%) were patients at Heidelberg University Hospital and 24 (38.1%) at Tübingen University Hospital. The mean age of the inserted restorations was 2.7 ± 1.9 years. Nineteen (19) patients (30.2%) had been treated for periodontitis and were participating in supportive periodontal therapy; 44 (69.8%) patients had no history of periodontitis; 42 patients (68.3%) were non-smokers, while 20 (31.7%) reported being smokers. The study population had a mean DMFT of 16.81 ± 5.28. Thirty-six patients (57.1%) stated that they used interdental brushes daily, while 27 (42.9%) did not use interdental brushes in their oral hygiene routine.

**Table 1:** Descriptive statistics of patient characteristics (n=63) expressed as mean ± SD for continuous variables as well as absolute and relative frequencies for categorical variables

Variable	Total (n = 63)
Age of restoration (in years)	2.70 ± 1.90
Usage of interdental brushes	36 (57%)
Smoker	20 (32%)
Periodontitis	19 (30%)
Decayed teeth	1.02 ± 2.47
Missing teeth	1.98 ± 2.62
Filled teeth	13.81 ± 4.56
DMFT index	16.81 ± 5.28
Bleeding on probing (in %)	18.96 ± 13.73
Plaque control record (in %)	53.69 ± 21.65
Gingival bleeding index (in %)	11.98 ± 9.76
Probing depth (in mm)	2.22 ± 0.53
Clinical attachment level (in mm)	2.40 ± 0.70

SD: standard deviation; DMFT: Decayed, Missing, Filled Teeth index.

### Gingival and Periodontal Condition

#### BOP

BOP did not occur significantly more often on test teeth compared to control teeth (OR = 1.633, CI: [0.731, 3.651], p = 0.232, Table 2). Patients of this cohort with treated periodontitis were not prone to increased gingival inflammation on teeth with subgingivally placed resin composite restorations, as the mixed effect models did not show a significantly higher amount of positive BOP in the subgroups “periodontitis: yes/no” on test and control teeth (OR = 0.688, CI: [0.268, 1.769], p = 0.438, Table 2).

**Table 2:** Results of the logistic mixed-effect models for local bleeding on probing (BOP) and local plaque control record (PCR), and of the linear mixed-effect models for local clinical attachment level (CAL)

		OR	Lower 95% CI	Upper 95% CI	p-value
Local BOP	Intercept	0.381	0.150	0.970	0.043
	Treated tooth	1.633	0.731	3.651	0.232
	Periodontitis: no	0.688	0.268	1.769	0.438
Local PCR	Intercept	3.822	0.403	36.273	0.243
	Treated tooth	1.000	0.127	7.855	1.000
	Periodontitis: no	0.658	0.056	7.741	0.740
	Treated tooth, parodontitis: no	0.597	0.049	7.334	0.687
		estimate	lower 95% CI	upper 95% CI	p-value
Local CAL	Intercept	3.123	2.802	3.445	0
	Treated tooth	0.421	0.057	0.785	0.027
	Periodontitis: no	-1.121	-1.498	-0.744	<0.001
	Treated tooth, periodontitis: no	-0.080	-0.516	0.356	0.720

OR: odds ratio; lower 2.5% CI and upper 97.5% CI stand for the confidence intervals of the odds ratio. BOP: No interaction term due to non-convergence.

#### GBI

The usage of interdental brushes had a significant influence on GBI (OR = 6.290, CI: [1.566, 11.013], p = 0.010, Table 3) on the test teeth. Regular use of interdental brushes leads to a decreasing probability of gingival bleeding. McNemar’s chi-squared test showed no significant increase of GBI on test teeth compared to control teeth (p = 0.228). As seen in Table 4, the relative frequency of gingival bleeding was higher on test teeth than on control teeth, and more gingival bleeding occurred in the subgroup of patients with a history of periodontitis in absolute terms. However, the mixed-effect models that were implemented to compare this inflammatory factor did not converge. Consequently, only the McNemar test was used for comparison between groups.

**Table 3:** Results of the regressions to evaluate possible influencing factors on local bleeding on probing (BOP), gingival bleeding index (GBI) on plaque control record (PCR) on treated teeth

		Estimate	2.5% CI	97.5% CI	p-value
Local BOP	Intercept	-12.929	-26.970	1.113	0.070
	Smoking: yes	5.363	-1.285	12.010	0.112
	Age of restoration (in years)	0.643	-1.058	2.344	0.452
	DMF-T	0.361	-2.054	0.976	0.245
	CAL	8.896	4.278	13.515	<0.001
	No use of interdental brushes	3.637	-2.495	9.769	0.240
Local GBI	Intercept	-1.193	-12.008	9.623	0.826
	Smoking	0.910	-4.210	6.031	0.723
	Age of restoration	0.087	-1.224	1.397	0.896
	DMF-T	-0.002	-0.476	0.472	0.993
	CAL	4.896	1.339	8.454	0.008
	No use of interdental brushes	6.290	1.566	11.013	0.010
Local PCR	Intercept	35.459	14.272	56.646	0.001
	Smoking	-9.480	-19.511	0.550	0.063
	Age of restoration	0.563	-2.004	3.129	0.662
	DMF-T	-0.058	-0.986	0.870	0.901
	CAL	3.983	-2.986	10.952	0.257
	No use of interdental brushes	0.279	-8.973	9.531	0.952

CI: confidence interval.

**Table 4:** Comparison of the relative frequencies (%) of “bleeding on probing (BOP): yes” and “bleeding on probing (BOP): no”, “gingival bleeding (GBI): yes” and “gingival bleeding (GBI): no”, of “dental plaque (PCR): yes” and “dental plaque (PCR): no”, probing depth (PD) in mm, and the clinical attachment level (CAL) in mm of test and control teeth with distinction between the subgroups “periodontitis: yes” and “periodontitis: no”

		Periodontitis: yes		Periodontitis: no	
		Test teeth	Control teeth	Test teeth	Control teeth
Local BOP	yes	42.1%	47.4%	52.3%	36.4%
	no	57.9%	52.6%	47.7%	63.6%
Local PCR	yes	73.7%	73.7%	72.7%	75.0%
	no	26.3%	26.3%	27.3%	25.0%
Local GBI	yes	47.4%	26.3%	13.6%	11.4%
	no	52.6%	73.7%	86.4%	88.6%
Local PD	in mm	3.0 ± 0.61	2.81 ± 0.57	2.50 ± 0.72	2.29 ± 0.54
local CAL	in mm	3.63 ± 0.87	3.15 ± 0.75	2.64 ± 0.84	2.33 ± 0.54

#### PCR

With regard to plaque accumulation (PCR), no significant difference was observed between test and control teeth (OR = 1.000, CI: [0.127, 7.855], p = 1.000, Table 2). Also, for patients with treated periodontitis, the mixed-effect models did not show a significant difference between test and control teeth. The regression model showed no significant influence of smoking status, age of the restoration, DMFT, CAL, or the usage of interdental brushes on the PCR (Table 3).

#### CAL

The results of the linear mixed-effect model showed that teeth with subgingivally placed composite restorations had on average 0.421-mm greater clinical attachment levels than control teeth, a statistically significant increase (CI: [0.057, 0.785], p = 0.027, Table 2). A history of periodontitis also had a significant influence, as patients without a prior history of periodontitis had a significantly lower CAL value (p < 0.001, Table 2). Considering only treated teeth, the clinical attachment level had a significant influence on BOP (p < 0.001) and GBI (p = 0.008, Table 3). The increase of CAL leads to an increasing probability of gingival bleeding (CI: [1.339, 8.454]) and an increasing probability of bleeding on probing (CI: [4.278, 13.515]).

#### Clinical quality

**Fig 2:**
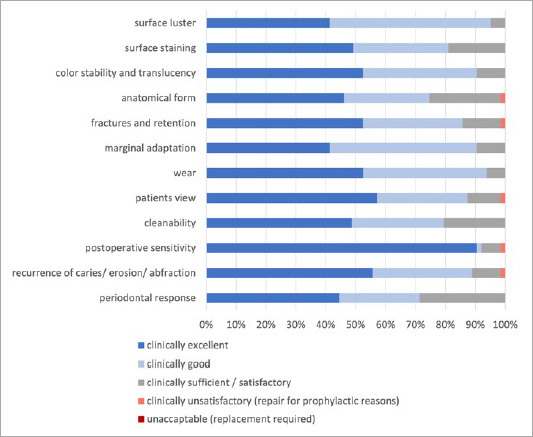
Evaluation of the FDI criteria on clinical quality of the subgingivally placed resin composite restorations (n = 63).

Figure 2 shows the results of the FDI criteria for the subgingivally placed resin composite restorations. In all categories, at least 70% of the restorations received high ratings. Only 5 categories showed the outcome “clinically unsatisfactory”. No examined restoration was unacceptable, consequently none had to be replaced after an observation period of 2.7 years.

## DISCUSSION

The results of this retrospective study demonstrate that deep subgingival direct restorations were not associated with increased periodontal or gingival inflammation over an observation period of approximately 3 years when regular interdental hygiene with interdental brushes was performed. In particular, there was no difference in bleeding on probing between test and control teeth (p = 0.232). The occurrence of bleeding on probing indicates an inflammatory condition at the base of the gingival sulcus and thus directly in the area of the marginal edge of the DME. The use of interdental brushes had an influence on the GBI (p = 0.010), which suggests that regular interdental hygiene, especially in the area of DME, is essential to maintain inflammation-free conditions. Deep subgingival defects are mainly caused by root decay and therefore already violate the supracrestal tissue attachment. Although reconstructed by a subgingival restoration, the extensive cavity entails a substantial loss of clinical attachment which can also influence periodontal and gingival inflammatory parameters. CAL on the test teeth was significantly increased compared to the control teeth in the periodontitis group (p = 0.027). Furthermore, increased CAL had a significant effect on BOP (p < 0.001) and GBI (p = 0.008). However, in this cohort, a prior history of periodontitis did not influence gingival inflammation signs or plaque accumulation on teeth with subgingivally placed composite restorations (Table 2).

The relatively small number of participants might also contribute to the effects measured here, and further studies are needed to gain more evidence. However, although the subject of our study is a recurrent problem, to the best of our knowledge, only one clinical study with a comparable number of examined restorations exists aside from ours.

The results of a 12-month controlled study on the effects of deep-margin elevation on periodontal health in 35 indirectly placed partial ceramic crowns were published in 2018 by Ferrari et al^17^ and are in line with our findings. At baseline and after 12 months, clinical inflammatory parameters (GBI, BOP, and PD) were measured. The study reported no initial sign of gingival inflammation or plaque occurrence in all subjects. Nevertheless, on sites with DME, BOP occurred significantly more often.^17^

In another clinical study on the response of periodontal tissue to subgingival composite restorations, Bertoldi et al^3^ enrolled 29 subjects with subgingival carious defects who underwent restorative post-endodontic therapy and were scheduled for a subsequent crown restoration. DMEs were performed on the teeth, and only cases in which the restoration margin did not violate the supracrestal tissue attachment were included, in combination with supportive periodontal care every 3 months. Histological samples showed no evidence of inflammatory processes in the DME area compared to control sites of the teeth.^3^

Compared to the present study, these two studies differ clearly in terms of sample size as well as the frequency and implementation of a structured oral prophylaxis program. Due to the retrospective character of the present study, a strict supportive prophylaxis program was not applied. Nevertheless, professional tooth cleaning and oral hygiene instructions were carried out regularly and according to the individual situation of the patient. Based on this difference in recall protocols and their strict exclusion criteria, significantly elevated inflammatory parameters may have been expected to occur in the present study. However, this was not observed. Although gingival bleeding was detected slightly more frequently than on the natural tooth surface, particularly in subjects with a prior history of periodontitis, the difference was not significant. We assume that the marginal quality of DMEs influences the extent of soft tissue irritation and biofilm adherence. Microbial biofilm on restorations placed in subgingival areas is responsible for the inflammatory response of the periodontal tissues. Hence, smooth, gapless composite resin margins seem to be tolerated without inflammatory irritation.^43^ The applied restorative method using a stepwise procedure with an initial DME and a subsequent direct composite restoration was apparently able to meet those requirements in the present investigation.

Another clinical study reported on the examination of 197 DME partial-indirect composite restorations in 120 subjects.^4^ The mean follow-up time was 57.7 months. The overall survival rate of the restorations was 95.5% after 10 years or longer. Those authors concluded that indirect restorations with DME showed good survival rates in the observed period of up to 12 years. Periodontal parameters were not recorded in the study, but the clinical quality criteria (FDI criteria) results of the baseline examination and at the last recall were presented. Here, a deterioration on the older restorations compared to the younger ones in all categories occurred, which the authors considered a physiological phenomenon of wear and aging.^4^ The present study investigated the restorations after a comparatively shorter observation period and showed predominantly very good or good clinical quality, which agrees with the findings by Bresser et al.^5^ This could indicate that the long-term results of DME with a direct composite restoration could also be positive. However, further long-term studies are needed.

**Fig 3:**
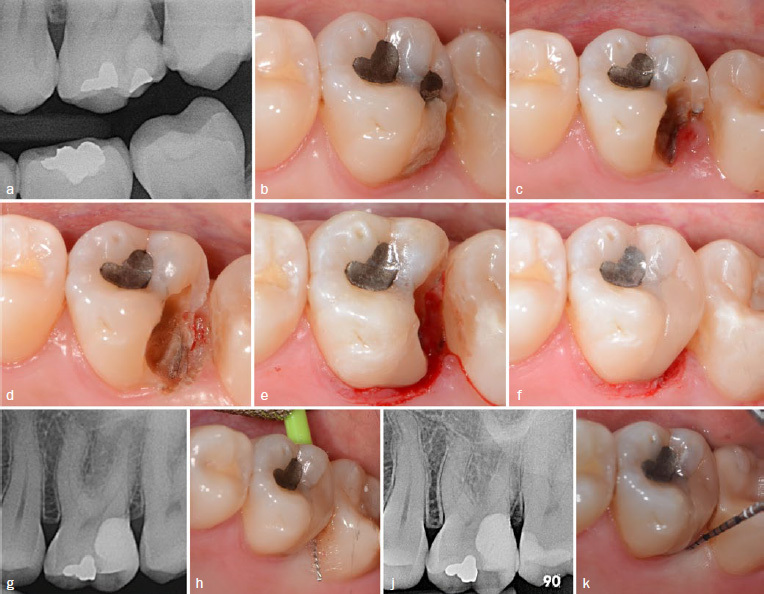
Clinical case of a 31-year-old female patient. The patient reported irritation-dependent pain in the region of tooth 26, with positive sensitivity and negative percussion tests. a) Radiographic initial situation of tooth 27 with secondary caries and an insufficient restoration proximal to the alveolar bone crest; b) clinically insufficient temporary restoration with chipping and insufficient margin in the proximal area; c) after removal of the temporary restorations, inflammed soft-tissue proliferation is visible in the area of the distal papilla; d) situation after caries removal and gingivectomy of the proliferated soft tissue; e) condition after finishing the restoration margins in DME: bleeding and contamination of the composite layer occurs; f) condition immediately after finishing and polishing of the restoration; g) radiographic follow-up after 31 months: the restoration is still close to the limbus alveolaris, no horizontal and/or vertical bone resorption detectable; h) clinical control after 31 months: no periodontal irritations are observed with excellent oral hygiene; j) radiograph of the situation after 9.5 years: no horizontal and/or vertical bone resorption visible; k) clinical situation after 9.5 years: PD is 2 mm without bleeding on probing.

It is noteworthy that 90.5% of the deep subgingival restorations were rated at least as “clinically good” in the “marginal adaptation” category, despite the more challenging fabrication process in the subgingival area. Since the placement of such a restoration poses many challenges (bleeding control, moisture isolation, reduced visibility of the cavity, insertion of composite without matrix), the practitioner should be well-trained. To improve the subgingival placement of composite material, techniques such as the matrix-in-a-matrix (M-i-M) technique have been described by Magne.^29^ In addition, the meticulous control of the deep margin in the technique-sensitive DME with a probe or – in some cases – radiographs are necessary. Overhanging margins can be associated with localized gingivitis, increase in PD, and interproximal bone loss.^14,40^ The high quality of the subgingival margin might be equally influenced by the “snowplow technique,” which can lead to more homogeneous restorations and margins.^38^ Choosing a standardized treatment procedure to produce smooth subgingival margins, as described by Frese et al,^19^ is recommended. Using DME, no alveolar bone loss was observed in the presented clinical case over 12 months. In Fig 3, a clinical case after DME is shown with an observation time of 9.5 years. Clinical and radiographic evaluation shows no signs of gingival or periodontal inflammation or alveolar bone loss (Fig 3). Jepsen et al^24^ stated that deep subgingival restorations are associated with inflammation and loss of periodontal tissue. However, they stated that there is no evidence on whether those effects on the periodontium are caused by biofilm, trauma, toxicity of dental materials, or a combination of these factors.^24^ Other studies support this claim, especially regarding toxicity of subgingivally placed adhesives and composite resin as used in this study.^13^

It should also be borne in mind that the alternative to DME is extraction or restoration with a crown of the affected tooth. In order to respect the supracrestal tissue attachment, surgical crown lengthening in the area of the corresponding crown margin or an orthodontic extrusion of the tooth is required.^10,16,20,36^ In case of extraction, an implant or fixed partial denture may be necessary. These treatment alternatives are not only invasive but also time-consuming and expensive. Therefore, DME in combination with a composite resin restoration can be an affordable and fast treatment option. However, DME should only be performed if the adhesive technique can be adequately performed in the area of the subgingival margin and smooth restoration margins can be created. This may depend on the experience of the practitioner, the depth of the defect, and its location (for example, carious lesions in a furcation). Furthermore, the findings of this study emphasize the importance of proximal dental hygiene, especially on those sites of the teeth with DME. If patients do not use interdental brushes in their dental cleaning regimen, the probability of gingival inflammation at these sites increases. These results are in line with Ercoli and Caton,^14^ who emphasize the importance of self-performed plaque control of patients with DME.

This study has several limitations. Firstly, calibration was only performed regarding the FDI criteria, and did not include periodontal parameters. Secondly, exact measurements of the distance between bone and the restoration margin were not taken. Furthermore, standardized radiographs were not available for all the restorations. Despite the present study’s retrospective nature, it increases the evidence on the clinical applicability of subgingival composite resin restorations, fabricated using a two-phase restoration process including DME and a subsequent direct composite restoration.

Despite the promising results of this study, it cannot be concluded that the supracrestal tissue attachment can generally be disregarded. Why violating the supracrestal tissue attachment leads to chronic inflammation in one patient and but not in another still requires further investigation. However, the presented results demonstrate that inflammation-free conditions are possible despite violation of the supracrestal tissue attachment, provided that the restorations have smooth margins. Furthermore, the use of interdental brushes in combination with regular participation in a preventive program should be ensured for long-term success of subgingivally placed direct composite restorations.

## CONCLUSION

No increased periodontal or gingival inflammation was observed on sites with subgingivally placed composite restorations over a mean observation period of approximately 3 years, although these sites showed higher levels of CAL due to subgingival defects. Regular interdental brush use was associated with less gingival inflammation. These findings suggest that DME is a valid treatment option for deep subgingival defects and is not associated with increased inflammation levels. Adequate oral hygiene supports the preservation of inflammation-free conditions.

## Clinical relevance:

Composite resin restorations can be placed subgingivally with smooth and irritation-free margins using deep-margin elevation. At the sites of the subgingivally placed composite resin restorations, inflammation-free conditions of the soft tissues are possible.
